# Revealing the Interaction Mechanism between *Mycobacterium tuberculosis* GyrB and Novobiocin, SPR719 through Binding Thermodynamics and Dissociation Kinetics Analysis

**DOI:** 10.3390/ijms25073764

**Published:** 2024-03-28

**Authors:** Xiaofei Qiu, Qianqian Zhang, Zhaoguo Li, Juan Zhang, Huanxiang Liu

**Affiliations:** 1 School of Pharmacy, Lanzhou University, Lanzhou 730000, China; qiuxf21@lzu.edu.cn (X.Q.); lzgcn@outlook.com (Z.L.); zhangjuan21@lzu.edu.cn (J.Z.); 2 Faculty of Applied Science, Macao Polytechnic University, Macao SAR, China; zhangqq@mpu.edu.mo

**Keywords:** tuberculosis, GyrB, novobiocin, SPR719, molecular dynamics (MD) simulations, binding mechanism, dissociation mechanism

## Abstract

With the rapid emergence of drug-resistant strains of *Mycobacterium tuberculosis* (Mtb), various levels of resistance against existing anti-tuberculosis (TB) drugs have developed. Consequently, the identification of new anti-TB targets and drugs is critically urgent. DNA gyrase subunit B (GyrB) has been identified as a potential anti-TB target, with novobiocin and SPR719 proposed as inhibitors targeting GyrB. Therefore, elucidating the molecular interactions between GyrB and its inhibitors is crucial for the discovery and design of efficient GyrB inhibitors for combating multidrug-resistant TB. In this study, we revealed the detailed binding mechanisms and dissociation processes of the representative inhibitors, novobiocin and SPR719, with GyrB using classical molecular dynamics (MD) simulations, tau-random acceleration molecular dynamics (τ-RAMD) simulations, and steered molecular dynamics (SMD) simulations. Our simulation results demonstrate that both electrostatic and van der Waals interactions contribute favorably to the inhibitors’ binding to GyrB, with Asn52, Asp79, Arg82, Lys108, Tyr114, and Arg141 being key residues for the inhibitors’ attachment to GyrB. The τ-RAMD simulations indicate that the inhibitors primarily dissociate from the ATP channel. The SMD simulation results reveal that both inhibitors follow a similar dissociation mechanism, requiring the overcoming of hydrophobic interactions and hydrogen bonding interactions formed with the ATP active site. The binding and dissociation mechanisms of GyrB with inhibitors novobiocin and SPR719 obtained in our work will provide new insights for the development of promising GyrB inhibitors.

## 1. Introduction

Tuberculosis (TB) is a chronic infectious disease caused by *Mycobacterium tuberculosis* (Mtb) infection, one of the neglected tropical diseases [1,2]. TB receives many resources in terms of funding, research focus, and international attention, so it is one the members of the “big three” (composed of Malaria and HIV) in infectious disease [3]. Globally, TB is the second leading cause of death from a single infectious agent after COVID-19, causing nearly twice as many deaths as HIV/AIDS [4]. According to the statistics from the World Health Organization (WHO), approximately one-fourth of the global population is infected with Mtb [4]. While tuberculosis is considered treatable, both first-line and second-line anti-tuberculosis drugs are currently facing significant drug resistance [5,6,7]. As the incidence of multiple drug-resistant tuberculosis (MDR-TB) strains continues to rise, there is an urgent need for new anti-tuberculosis drugs to replace existing ones. One strategy for identifying potential new antibiotics is to re-examine attractive but relatively underexplored targets.

The DNA gyrase subunit B (GyrB) is a clinically validated target that has not been extensively developed [8]. DNA gyrase, a highly conserved bacterial topoisomerase, consists of the GyrA and GyrB subunits, forming a heterotetramer (A_2_B_2_) of disparate origins. It plays a crucial role in maintaining and regulating the DNA’s topological structure within the cell, essential for bacterial DNA replication. The GyrA subunit is involved in DNA cleavage and rejoining, while the GyrB subunit, containing an ATPase active site, binds and hydrolyzes ATP to generate the energy required for introducing negative supercoils necessary for DNA replication [9,10]. Fluoroquinolone drugs targeting GyrA have been extensively studied as anti-tuberculosis agents [11]. However, mutations in the GyrA domain have reduced the effectiveness of these drugs, leading to increased resistance [12]. Consequently, attention has shifted to GyrB’s ATPase. GyrB has shown bactericidal activity against Mtb, and its ATP binding site is highly conserved, reducing the likelihood of developing spontaneous resistance to ATP competitive inhibitors. Resistance studies have also shown no cross-resistance between GyrB inhibitors and fluoroquinolone drugs [13]. Moreover, GyrB forms a homodimer in eukaryotic cells, a structural difference that allows its inhibitors to act more selectively on pathogens [14]. Given these advantages, research on GyrB inhibitors has attracted significant interest.

**Figure 1:**
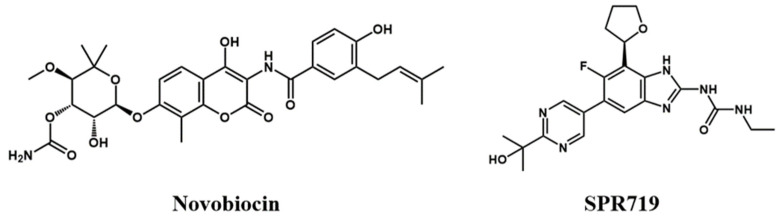
Two representative GyrB inhibitors, novobiocin and SPR719.

Several scaffolds with inhibitory activity against Mtb GyrB have been reported, such as pyrrolamides [15,16,17], benzimidazoles [18,19], thiazolopyrimidinones [20,21], indolylidine nitrothiazoles [22], aminopyridines [23], aminopyridazines [24], and others. However, to date, only novobiocin (Figure 1), an antibiotic targeting GyrB, has been approved for human use, and its efficacy has been confirmed in pre-clinical and clinical trials [25]. Novobiocin was first approved in 1964, primarily used for the treatment of severe infections caused by methicillin-resistant Staphylococcus aureus (MRSA). However, due to safety concerns related to its toxicity, it was required by the FDA to be withdrawn from the market in 2009 [26]. Nevertheless, novobiocin remains the only clinically validated GyrB inhibitor and has been shown to possess antibacterial activity against Mtb [13]. In addition, the second-generation benzimidazole compound SPR719, discovered through structure optimization and metabolism transfer strategies, has demonstrated bactericidal activity against various drug-sensitive and drug-resistant strains. It has shown excellent efficacy in a mouse chronic Mtb infection model, with a relatively low spontaneous resistance frequency [18,19,27], and is currently in Phase II clinical trials [28]. There are currently no drugs available on the market that target the GyrB ATP binding site. Therefore, gaining a comprehensive understanding of the molecular interactions between these representative inhibitors and GyrB holds significant practical importance for the future design and improvement of more potent GyrB inhibitors.

The crystal structure of Mtb GyrB in complex with adenosine 5′-(β, γ-imino) triphosphate (AMPPNP) was reported in 2013 [29]. The emergence of these crystal structures has provided valuable information for understanding the critical interactions between the protein’s active pocket and inhibitors. This information can be used not only to study the binding thermodynamics of GyrB with inhibitors but also serve as a good starting point to elucidate the detailed molecular mechanisms of inhibitor dissociation from the protein binding pocket. Based on this information, this study initially explored the binding thermodynamics of novobiocin and SPR719 with GyrB through classical molecular dynamics (MD) simulations. The Molecular Mechanics-Generalized Born Surface Area (MM-GBSA) method was used to predict the binding free energy between protein and ligands. The results indicate that these two inhibitors primarily bind to GyrB through electrostatic and van der Waals interactions. Residue-based energy decomposition identified some key residues, such as Asn52, Asp79, Arg82, Lys108, Tyr114, and Arg141, that have significant energy contribution to the inhibitors binding. Simultaneously, tau-random acceleration molecular dynamics (τ-RAMD) simulations were used to explore the dissociation pathways of the inhibitors, and steered molecular dynamics (SMD) simulations were used to further characterize the transition states during the dissociation process for both inhibitors. Through these simulations and calculations, insights into the binding and dissociation mechanisms of novobiocin and SPR719 with GyrB have been provided. This information offers valuable guidance for the rational design of GyrB ATPase inhibitors.

## 2. Results

### 2.1. Electrostatic Interactions and van der Waals Interactions Are the Primary Driving Forces for the Binding of GyrB Inhibitors

**Figure 2:**
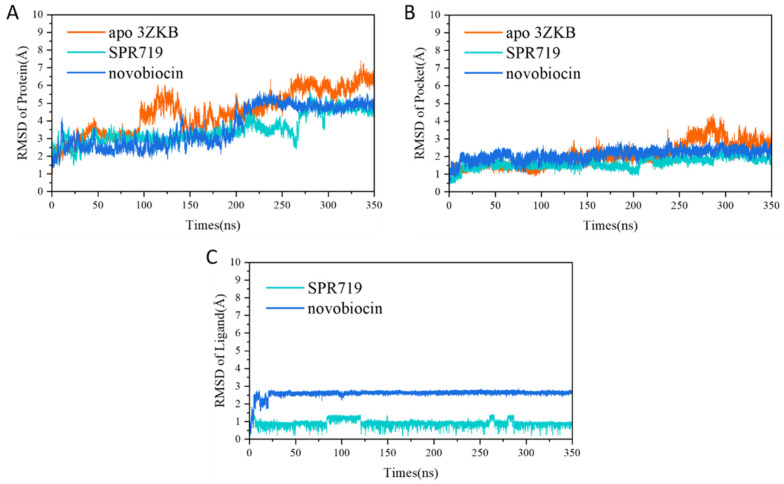
(**A**) Time evolution of RMSD for the protein’s Cα atoms; (**B**) time evolution of RMSD for the residues of the binding pocket (within 5 Å of the ligand); (**C**) time evolution of RMSD for the non-hydrogen atoms of the ligand.

First, to assess the system’s stability, the root-mean-square deviation (RMSD) of the Cα atoms of the protein, the residues within 5 Å of the binding pocket, and the non-hydrogen atoms of the ligand over the simulation were monitored. As depicted in Figure 2A, the RMSD fluctuations of the entire protein are relatively large, which may be due to fluctuations in some flexible loop regions. However, Figure 2B shows that whether in the apo system or the complex system, the RMSD fluctuation of the binding pocket is small, within 3 Å, especially the binding of inhibitors makes the residues of the binding pocket more stable (RMSD stable at around 2 Å). Additionally, it can be seen from Figure 2C that the RMSD value of novobiocin is larger than that of SPR719, indicating that the conformation of novobiocin has changed significantly. However, the conformations of both inhibitors stabilized during the simulation and could be used for subsequent analysis.

**Figure 3:**
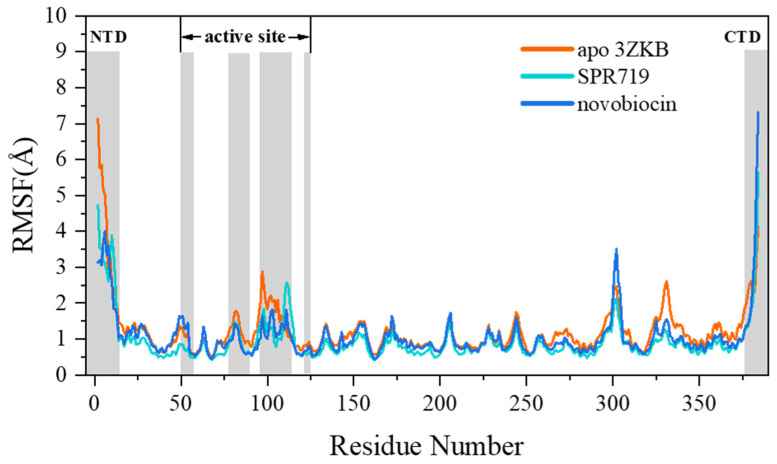
RMSF of Cα atoms of protein residues in the three systems.

Subsequently, the root-mean-square fluctuation (RMSFs) for the last 50 ns of the three systems were calculated to investigate the flexibility of residues. As shown in Figure 3, it can be observed that residues in all three systems exhibit similar trends in RMSF values. In particular, the residues in the protein–ligand complex systems display even more similar fluctuations. Additionally, not only do residues located far from the binding site, such as at the N-terminus domain (NTD) and C-terminus domain (CTD) of the protein, show higher flexibility, but residues in sequences 77–89 and 96–114 at the GyrB active site also exhibit significant flexibility. Moreover, the apo system at the binding site exhibits greater fluctuations compared with the two complex systems, indicating that the inhibitors play a role in stabilizing the residues at the protein’s binding site.

The binding free energies were calculated using the MM-GBSA method, which has been widely used in numerous studies to explore the detailed interactions between two representative inhibitors and the target from an energetic perspective [30,31,32]. For each system, 1000 snapshots were extracted from the last 50 ns trajectories to calculate the average binding free energy. As shown in Table 1, the binding free energy of novobiocin with GyrB is −24.34 kcal/mol, and that of SPR719 is −26.81 kcal/mol. For ease of comparison, we converted the experimentally obtained Ki value [18,33] into binding free energy. As shown in Table 1, the experimental binding energies (Δ*G*_exp_) of novobiocin and SPR719 are −10.82 kcal/mol and −11.04 kcal/mol, respectively. It can be seen that the ranking of calculated binding free energy of novobiocin and SPR719 is well consistent with the experimental value. By comparing the contributions of each energy term, it is evident that the electrostatic interaction term (∆*E*_ele_) is crucial for ligand binding, with novobiocin and SPR719 showing values of −48.72 kcal/mol and −62.04 kcal/mol, respectively. Furthermore, contributions from the van der Waals interaction term (∆*E*_vdw_) and the nonpolar solvation-free energy term (∆*G*_nonpolar_), both exceeding −59.8 kcal/mol for each inhibitor, underscore the significance of hydrophobic interactions in stabilizing the ligands within the binding pocket. Therefore, ∆*E*_ele_, ∆*E*_vdw_, and ∆*G*_nonpolar_ are the primary driving forces for inhibitor binding to the target, facilitating the stability of small molecules in the binding site. Additionally, the polar solvation energy term (∆*G*_polar_) for both systems showed positive values, indicating an unfavorable contribution to the binding of both inhibitors. This is attributed to the large volume of the ligand-binding pocket, resulting in extensive exposure to solvent.

### 2.2. Residue Energy Decomposition Revealed the Key Residues for Inhibitor Binding to GyrB

**Figure 4:**
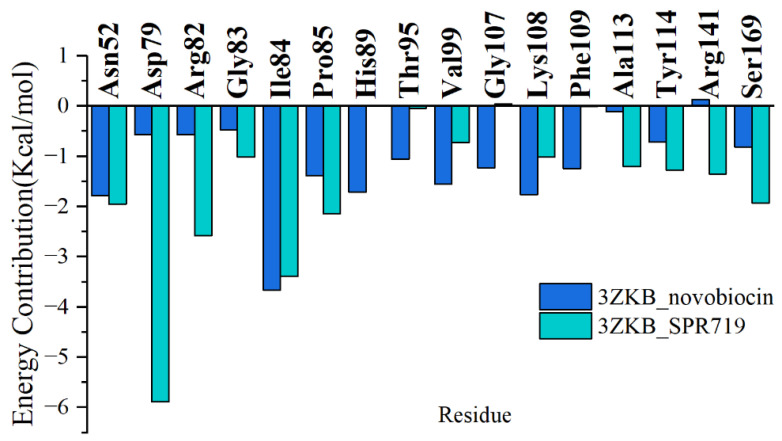
Energy contribution spectrum of key residues for binding of two inhibitors.

To further identify the residues playing a key role in the binding process of representative inhibitors with GyrB, the method of residue energy decomposition was used to decompose the binding free energy onto each residue, as shown in Figure 4. Based on the contributions of each residue to the interaction energy, we identified nine residues that contribute to the binding of novobiocin and SPR719: Asn52, Asp79, Arg82, Ile84, Pro85, Val99, Lys108, Tyr114, and Ser169, with free energy contributions ranging from −0.56 to −5.89 kcal/mol.

**Figure 5:**
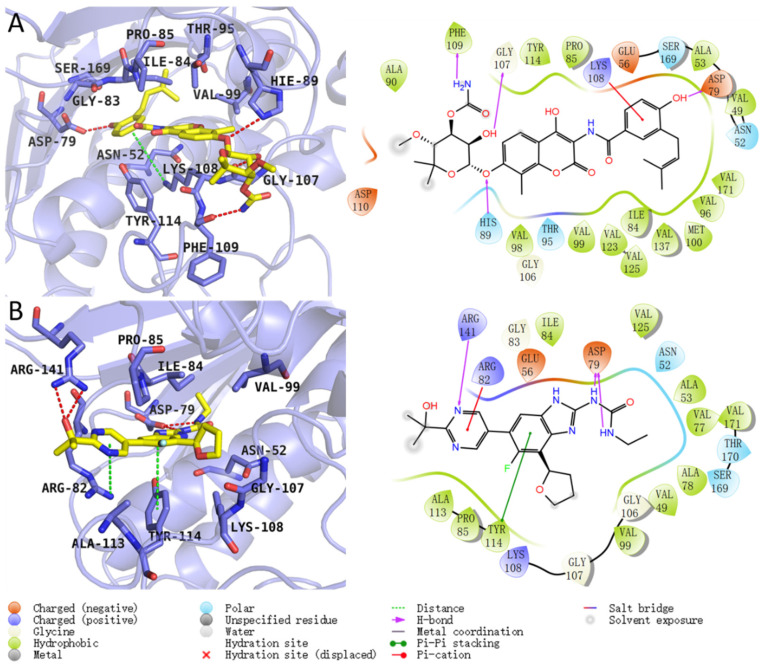
Detailed 3D and 2D binding modes of representative conformations of (**A**) novobiocin and (**B**) SPR719 binding to the ATP site of GyrB. In the 3D binding mode images, the protein’s secondary structure is depicted in cartoon form, key residues involved in ligand binding are shown as purple sticks, and the inhibitors are represented as yellow sticks. Red dashed lines represent hydrogen bond interactions, and green dashed lines represent π interactions. In the 2D binding mode images, purple arrows represent hydrogen bond interactions, red lines indicate π–cation interactions, and green lines represent π–π interactions.

To further analyze the detailed binding modes of the two inhibitors to GyrB, cluster analysis was performed based on the equilibrium trajectories of the last 50 ns of each system. In the cluster analysis, the RMSD of the complexes was used as the metric and the clustering cutoff was set to 4.0 Å. As shown in Table S1, both systems have three clustering centers, among which cluster1 accounts for 83.6% and 90% for novobiocin and SPR719, respectively. Therefore, the structure located at the center of cluster1 was extracted as the representative structure of each system. The detailed binding modes between the two inhibitors and GyrB are shown in Figure 5. In the novobiocin system, the benzene ring on the inhibitor can form a π–cation interaction with Lys108, leading to a higher energy contribution from the Lys108 residue. Additionally, the strongly polar amino and hydroxyl groups on the inhibitor serve as hydrogen bond donors, interacting with residues Asp79, Gly107, and Phe109 to form hydrogen bonds. The amino group on the imidazole ring of His89 also acts as a hydrogen bond donor, forming hydrogen bonds with the inhibitor. These hydrogen bonds significantly stabilize the binding between novobiocin and GyrB. Furthermore, the tail of the inhibitor can establish strong van der Waals interactions with hydrophobic residues such as Val49, Ala53, Ile84, Pro85, Val96, Val99, Met100, and Val125. Among these residues, Asn52, Ile84, Pro85, His89, Val99, Gly107, Lys108, and Phe109 make substantial energy contributions to the stable binding of novobiocin. In the SPR719 system, the Asp79 residue contributes the most to the energy. Upon analyzing the binding mode, this is primarily due to the formation of two hydrogen bonds between the carboxyl oxygen atom of the Asp79 residue and two amino hydrogen atoms on the urea group of SPR719. Additionally, the nitrogen atom of the pyrimidine ring of SPR719 not only forms a hydrogen bond interaction with Arg141 but also engages in π–cation stacking interaction with Arg82. The benzene ring of SPR719 also forms a π–π interaction with Tyr114. Furthermore, the benzene ring of SPR719 can establish strong van der Waals interactions with surrounding hydrophobic residues (Ile84, Pro85, Ala113, Tyr114). The tail of SPR719 extends into a hydrophobic pocket formed by residues such as Val49, Ala53, Val77, Ala78, Val99, and Val125. These interactions collectively stabilize the binding between SPR719 and GyrB. Moreover, residues such as Asn52, Arg82, Ile84, Pro85, Ala113, Tyr114, and Arg141, as shown in Figure 4, make significant energy contributions to the binding of SPR719.

Comparing the binding modes of the two inhibitors with GyrB, it is evident that the binding modes of both inhibitors are highly similar. Firstly, both of them deeply penetrate into the binding pocket, and the phenyl ring or tail of each inhibitor binds to a hydrophobic pocket formed by nonpolar amino acid residues, including Val49, Val53, Ile84, Pro85, Val99, Phe109, Ala113, Tyr114, and Val125, leading to strong van der Waals interactions. Secondly, both inhibitors form hydrogen bond interactions with Asp79, and the hydrogen bond occupancy with Asp79 remains stable for both inhibitors. As shown in Table 2, the hydrogen bond occupancy with Asp79 for novobiocin and SPR719 is 99% and 75%, respectively, further emphasizing the significance of the interaction with the Asp79 residue for inhibitor binding. Finally, the π–cation interactions between the phenyl ring or nitrogen-containing ring deeply within the pocket and specific amino acid residues in the binding pocket are also crucial.

The detailed interactions between proteins and inhibitors, as well as the identification of hotspot residues at the protein-inhibitor binding interface, can provide valuable information for the discovery and design of GyrB inhibitors. Therefore, gaining a comprehensive understanding of the interactions between GyrB and inhibitors and determining the key residues at the binding interface is crucial. According to the results of molecular dynamics simulations, the aromatic rings and hydrophobic centers play a crucial role in protein-inhibitor binding. Simultaneously, the presence of polar centers in the inhibitors, particularly their hydrogen bond interactions with the Asp79 residue, is of paramount importance. This further confirms that van der Waals interactions and electrostatic interactions are the primary driving forces for inhibitor binding. The current research findings contribute to a deeper understanding of the detailed interactions between GyrB and representative inhibitors, guiding the future design of GyrB inhibitors. Additionally, in subsequent virtual screenings targeting GyrB, compounds capable of forming hydrogen bond interactions with the Asp79 residue will be prioritized as potential lead compounds.

### 2.3. tau-RAMD Simulations Revealed That the Dissociation Pathways of Novobiocin and SPR719 Were Mainly from the ATP Channel of GyrB

**Figure 6:**
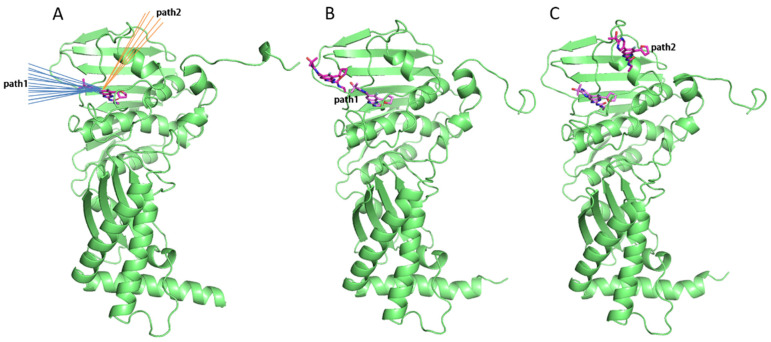
(**A**) Schematic representation of the two possible dissociation pathways for the inhibitors (path1 and path2). The blue lines represent path1, and the orange lines represent path2. (**B**) Schematic depiction of SPR719 dissociating along path1. (**C**) Schematic depiction of SPR719 dissociating along path2. Proteins are shown as green cartoons and ligands are shown as magenta sticks.

The tau-RAMD (τ-RAMD) simulations provide an effective means to rapidly identify the dissociation pathways of inhibitors. We utilized this method to investigate the potential dissociation pathways for novobiocin and SPR719 from the GyrB ATP binding site. As shown in Figure 6, we identified two possible dissociation pathways. Specifically, path1 represents a dissociation pathway along the ATP channel, while path2 involves dissociation by moving the loop region connected to helix5 upwards, creating space for the inhibitor to dissociate. Additionally, we classified and statistically analyzed the dissociation directions of 100 simulated trajectories for both inhibitors (as detailed in Table S2). The statistical results indicate that the primary dissociation pathway for these two inhibitors is path1, involving dissociation from the ATP channel, with path2 being rarely observed.

### 2.4. SMD Simulations Identified the Intermediate States in the Dissociation Process and the Key Amino Acids Involved

**Figure 7:**
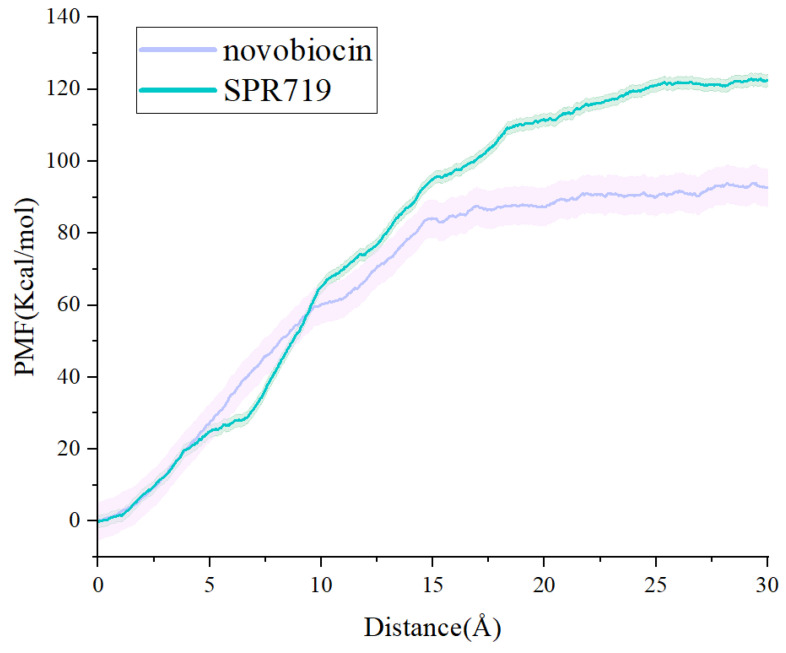
PMF curves for the dissociation of two inhibitors, novobiocin and SPR719, from the ATP channel.

In order to investigate the dissociation kinetics of inhibitors, we conducted steered molecular dynamics (SMD) simulations to study the dissociation process of novobiocin and SPR719 from the ATP binding pocket of GyrB. First, based on the main dissociation pathways obtained from τ-RAMD simulations, we defined the reaction coordinates for the dissociation of the two inhibitors along the ATP channel. Secondly, to ensure the use of a rigid spring approximation in the SMD simulations, we evaluated different stretching velocities (0.01, 0.005, 0.008, and 0.0008 Å/ps) and elastic constant (40, 50, 60, and 70 kcal/mol·Å^−2^). The results showed that a stretching velocity of 0.008 Å/ps and an elastic constant of 60 kcal/mol·Å^−2^ met the requirements for maintaining a hard spring state throughout the entire SMD simulation. Based on the rigid spring pulling, we employed 10 parallel SMD simulation trajectories in conjunction with the Jarzynski equation to calculate the change in the potential of mean force (PMF) for both sets of systems (as shown in Figure 7). The results indicate that the PMF for novobiocin and SPR719 is 96.40 ± 5.22 kcal/mol and 122.35 ± 1.71 kcal/mol, respectively. This trend aligns with experimental values of Ki for small molecules reported in the literature [18,33] as well as the calculated binding free energies using the MM-GBSA method (Table 1). It suggests that SPR719 has to overcome a greater binding free energy barrier during the dissociation process compared to novobiocin.

**Figure 8:**
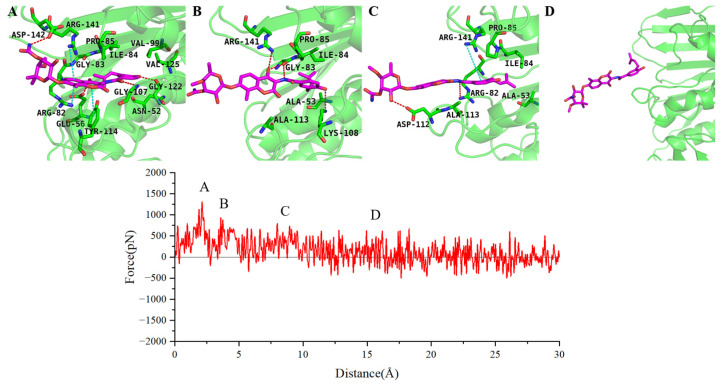
The force profile of novobiocin over the reaction coordinates along ATP channel, where (**A**–**D**) represent the structures of novobiocin at corresponding points along the reaction coordinate.

To further analyze the specific dissociation pathways of the two inhibitors, we selected representative SMD trajectories from the 10 dissociation trajectories to examine the forces experienced by the two inhibitors during dissociation and the intermediate conformational states in their dissociation process. During the dissociation along the ATP channel, novobiocin exhibits three distinct intermediate states (as shown in Figure 8). In the first intermediate state, novobiocin forms stable hydrogen bonds with Glu56, Gly107, Gly122, and Asp142. The benzimidazole ring on the inhibitor also engages in π interactions with Arg82, Arg141, and Tyr114, hindering the dissociation of the inhibitor at this point (Figure 8A). In the second intermediate state, the hydrophobic tail of novobiocin detaches from the pocket and forms new hydrogen bonds with Gly83, Lys108, and Arg141. Simultaneously, the inhibitor experiences hindrance from van der Waals interactions with hydrophobic residues near the pocket, including Ala53, Ile84, Pro85, and Ala113, during the dissociation process (Figure 8B). In the third intermediate state, the inhibitor forms new hydrogen bonds with Arg82 and Asp112. The π–cation interaction between the electron-rich benzene ring at the inhibitor’s tail and Arg141 hinders rapid dissociation from the binding site (Figure 8C). Finally, the force acting on novobiocin drops to near zero and fluctuates, indicating that novobiocin has completely departed from the GyrB binding site (Figure 8D).

**Figure 9:**
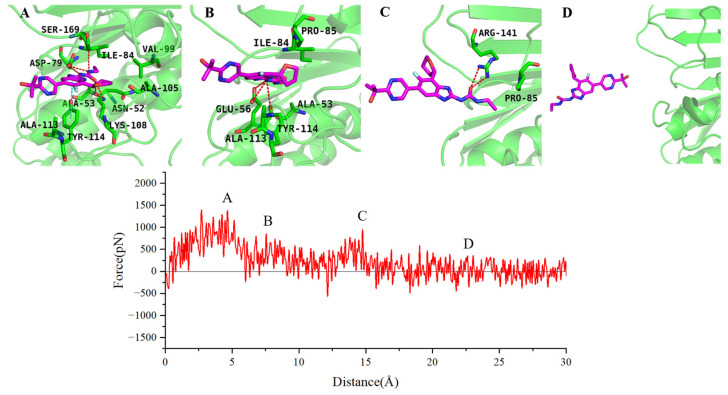
The force profile of SPR719 over the reaction coordinates along ATP channel, where (**A**–**D**) represent the structures of SPR719 at corresponding points along the reaction coordinate.

SPR719 and novobiocin share some similarities in the dissociation process. Moreover, they both exhibit three distinct intermediate states during dissociation along the ATP channel (as illustrated in Figure 9). For the SPR719 system, the first intermediate state is primarily hindered by stable hydrophobic interactions formed at the binding site by Val49, Ala53, Ile84, Val99, and Ala105. Simultaneously, SPR719 forms hydrogen bond interactions with Asn52, Asp79, Lys108, and Ser169 during the dissociation process. The benzene ring of SPR719 also engages in π–π interactions with Tyr144. These protein–inhibitor interactions collectively impede the dissociation of SPR719 from the binding site (Figure 9A). In the second intermediate state, similar to novobiocin, the hydrophobic tail of SPR719 detaches. The hydrogen atom on the terminal amino group can form hydrogen bonds with the side chains of Glu56 and Tyr114. Additionally, hydrophobic residues such as Pro85, Ala113, and Tyr114 near the dissociation exit hinder the dissociation of SPR719 by forming van der Waals interactions with the benzene ring (Figure 9B). In the third intermediate state, the inhibitor dissociates from the binding pocket, and the oxygen atom on its urea group forms a hydrogen bond with Arg141 (Figure 9C). Approximately 20 Å later, SPR719 completely dissociates from the dissociation channel (Figure 9D). While the main dissociation pathway of SPR719 aligns with novobiocin, SPR719 binds more deeply into the pocket, allowing it to form more interactions with surrounding residues such as Asn52, Asp79, Ala113, and Ser169. Consequently, the PMF for SPR719 dissociation is greater than that for novobiocin.

**Figure 10:**
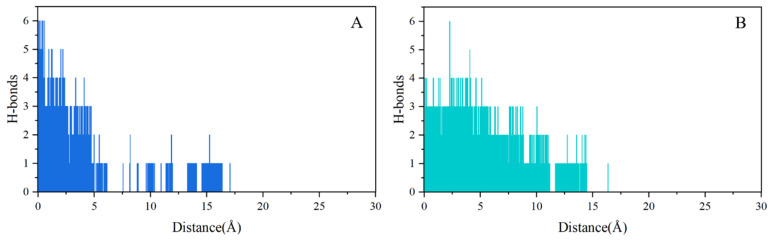
Changes in the number of hydrogen bonds during the dissociation process for both systems: (**A**) GyrB-novobiocin system, (**B**) GyrB-SPR719 system.

The key hydrogen bonds formed during the dissociation of inhibitors from the ATP-binding site of GyrB represent the main interactions between the inhibitors and the target. Therefore, we investigated the change in the number of hydrogen bonds in both systems during the dissociation process. As shown in Figure 10, it can be observed that novobiocin exhibits a gradual pattern of hydrogen bond rupture, with the overall number of hydrogen bonds decreasing to 1 and 0 after 5 Å. In contrast, SPR719 forms more persistent hydrogen bond interactions, still maintaining 2 to 3 hydrogen bonds after 5 Å. This means that the hydrogen bond interactions established by SPR719 along the ATP channel during the dissociation process are stronger and more numerous, making it more difficult for the inhibitor to dissociate. Furthermore, monitoring the number of hydrogen bonds indicates that hydrogen bonds play a crucial role in the inhibitor dissociation process.

Through the analysis of the interactions between the two inhibitors and the receptor during the dissociation process, we have concluded that SPR719 forms relatively strong hydrophobic and hydrogen bond interactions with GyrB. Therefore, it needs to overcome a greater free energy barrier to dissociate from the target protein. Furthermore, the key amino acid residues that affect the dissociation of both inhibitors are primarily Asn52, Glu56, Asp79, Arg82, Ile84, Pro85, Gly107, Lys108, and Arg141. These findings align with the results obtained from the analysis of binding kinetics.

## 3. Discussion

In this study, we employed a range of computational methodologies, including molecular dynamics (MD) simulation, Molecular Mechanics-Generalized Born Surface Area (MM-GBSA) calculations, τ-Random Accelerated Molecular Dynamics (τ-RAMD) simulations, and steered molecular dynamics (SMD) simulations, to comprehensively investigate the mechanisms of binding and dissociation between Mtb GyrB and its inhibitors novobiocin and SPR719. Our results suggest that the primary driving forces responsible for the binding of inhibitors to the target protein are electrostatic interactions and van der Waals forces, which is consistent with the findings of Tambe and colleagues [34]. The findings from residue energy decomposition and binding mode analysis underscore the importance of Asn52, Asp79, Arg82, Ile84, Pro85, and Arg141 as essential residues for the stable binding of inhibitors at the GyrB ATP binding site. Specifically, Asp79 is highlighted for its ability to form hydrogen bonds with inhibitors. This aligns with the results of Kamsri et al.’s [35] MD simulation study on GyrB inhibitors of thiadiazole derivatives, which also identified Asn52, Asp79, Arg82, Ile84, and Arg141 as key residues contributing significantly to energy residue and emphasizing their critical role in inhibitor binding. The study conducted by Gl et al. [36] identified some novel drug candidates targeting GyrB through drug repurposing. Through molecular docking and subsequent molecular dynamics (MD) simulation analysis, it was observed that the inhibitor established a stable interaction with the crucial residues Asn52, Asp79, and Arg82 of the ATP binding site. Additionally, a further examination of the research by Arévalo et al. [37] validated that the inhibitor frequently engaged in hydrogen bonding with Asp79 during the MD simulation. These results support and augment our comprehension of the primary forces governing the inhibitor–GyrB ATP binding and the essential residues implicated, thus validating the precision and efficacy of our methodology. The identification of these key residues can provide an important theoretical basis for the discovery and design of further GyrB inhibitors.

In the context of drug development, understanding the dissociation kinetics of drugs and targets is crucial. Despite this, the dissociation pathway and associated information regarding GyrB inhibitors remain undisclosed. Consequently, this study utilized τ-RAMD and SMD simulations to elucidate the dissociation pathway of novobiocin and SPR719 from the Mtb GyrB ATP binding site. Initial findings from the τ-RAMD simulations indicate that both inhibitors primarily dissociate via the ATP channel. SMD simulations further elucidated the intricate dynamics of inhibitor dissociation, highlighting the necessity for inhibitors to surmount hydrophobic interactions with residues such as Asn53, Ile84, Pro85, Val99, and Ala113 within the GyrB ATP active site, in addition to forming hydrogen bonds with Glu56, Aap79, Lys108, and Arg141. The transient nature of these interactions during dissociation offers significant insights into the rational design of drugs based on structural considerations. Overall, this research not only aids in a deeper understanding of the binding and dissociation mechanisms between GyrB and its inhibitors but also lays a solid theoretical foundation for designing and developing novel GyrB inhibitors with higher affinity.

## 4. Materials and Methods

### 4.1. Molecular Docking

Because the structure of the complex of Mtb GyrB with novobiocin and SPR719 has not been solved yet, the complex for MD simulation was obtained by molecular docking based on the complex of Mtb GyrB with AMPPNP (PDB ID: 3ZKB). Molecular docking was implemented by the Glide program in the Schrödinger 2021 software package. The crystal structure of the Mtb GyrB complex with AMPPNP was obtained from the RCSB database https://www.rcsb.org/ (accessed on 21 May 2023). The Protein Preparation Wizard module of Schrödinger 2021 was used to prepare the protein structure, such as removing the crystallographic water molecules and adding missing hydrogen atoms. The Receptor Grid Generation module was employed to define the binding site, which is centered on the ligand AMPPNP. The LigPrep module was then used to prepare the ligands, which included generating three-dimensional conformations for the small molecules, determining protonation states, and generating isomers. Firstly, in order to evaluate the accuracy of molecular docking methods in predicting binding conformations of ligands, the ligand AMPPNP was extracted from the crystal structure 3ZKB and re-docked to the binding pocket using both Standard Precision (SP) and Extra Precision (XP) modes. Upon calculating the root-mean-square deviation (RMSD) between the re-docked conformations and the original crystallographic conformation, we found that the RMSD from SP docking was 1.17 Å, whereas the RMSD from XP docking was 1.92 Å. Therefore, the SP docking was performed for novobiocin and SPR719, which provided the initial structures for MD simulations of the two systems.

### 4.2. System Preparation

Before performing molecular dynamics simulations, we initially used Gaussian 09 software to calculate the electrostatic potentials of the two inhibitors. Subsequently, RESP [38,39] charge fitting was conducted using the Antechamber program within the AMBER 20 software suite. The Parmchk module was further utilized to generate parameters for the inhibitors. The tleap module was employed to create the topological and coordinate files for the complexes. The general AMBER force fields ff14SB [40] and GAFF [41,42] were used to describe the protein and inhibitors, respectively. Finally, the complexes were placed in a cubic water box based on the TIP3P solvent model, with the distance between the complex and the box boundary set to 10 Å. Na^+^ ions were added to each system to neutralize the entire system.

### 4.3. Classical Molecular Dynamics Simulations

All MD simulations were performed using the Amber 20 program [43]. Initially, the first 2500 steps employed the steepest descent method, followed by an additional 2500 steps using the conjugate gradient method for energy minimization. Subsequently, each system was heated from 0 K to 300 K in the NTV ensemble, with a constraint on the complex using a force constant of 2 kcal/(mol·Å^2^) [44]. Following this, four equilibration steps were carried out in the NPT ensemble [45], applying constraints on the complex that decreased from 2.0 to 1.0 to 0.5 to 0.1. Next, to eliminate atomic collisions and relax the entire system, a 500 ps unrestrained equilibrium MD simulation was conducted. Finally, each system underwent a 350 ns molecular dynamics simulation. During the simulations, the Particle Mesh Ewald (PME) algorithm was employed to handle long-range electrostatic interactions [46], and the SHAKE algorithm [47] was used to constrain the vibrations of covalent bonds involving all hydrogen atoms. The time step was set to 2 fs. Additionally, all cartoon plots were generated using PyMOL 2.7.

### 4.4. τ-Random Accelerated Molecular Dynamics (τ-RAMD) Simulation

The τ-Random Accelerated Molecular Dynamics (τ-RAMD) is an extension of the RAMD method, designed to facilitate ligand dissociation from the protein binding pocket within a limited time scale [48,49,50]. The main advantage of τ-RAMD is its ability to automatically search for potential dissociation pathways near the binding pocket without the need for predefined dissociation directions or extensive parameter fitting [51]. In this study, τ-RAMD simulations were conducted using the NAMD program [52]. Initially, 10 ns of conventional molecular dynamics simulations were performed to obtain the initial structures required for τ-RAMD simulations. Five initial structures were extracted from the simulation trajectories of each system (one frame every 2.5 ns) for subsequent τ-RAMD simulations. Additionally, the magnitude of the random force was set to 25 kcal/mol. Each initial structure underwent 20 parallel dissociation simulations, where the magnitude of the random force remained constant, but the direction of the force changed randomly to enhance the reliability of the simulations. Finally, each system obtained a total of 100 dissociation trajectories.

### 4.5. Steered Molecular Dynamics Simulations

Steered molecular dynamics simulations (SMD) [53] are an enhanced sampling technique widely employed to explore the intermediate states of inhibitor dissociation from protein binding pockets [50,54,55,56,57,58]. SMD simulations apply external forces to one or more atoms in the ligand, guiding it to dissociate in a specified direction. This direction is determined by the primary dissociation pathway obtained from τ-RAMD simulations, setting the reaction coordinate of the inhibitor to follow the ATP channel dissociation direction. In this study, the constant velocity pulling SMD simulation method was used. Simulation parameters were adjusted to ensure a rigid spring during constant velocity stretching. Initially, the elastic constant k was set to 60 kcal/mol·Å^−2^, and different stretching velocities v were tested as follows: 0.01, 0.005, 0.008, and 0.0008 Å/ps (Figure S1). Subsequently, with the stretching velocity v fixed at 0.008 Å/ps, the elastic constant k was adjusted to 40, 50, 60, and 70 kcal/mol·Å^−2^ (Figure S2). The results showed that a spring constant (k) of 60 kcal/mol·Å^−2^ and a pulling velocity (v) of 0.008 Å/ps met the requirements for a stiff spring (Figure S3). Additionally, to prevent the translation and rotation of the protein during simulation, position restraints were applied to certain residues away from the binding pocket.

In order to examine the free energy barrier linked to the dissociation of the two inhibitors from the ATPase binding site of GyrB, we reconstructed the potential of mean force (PMF) profiles from SMD simulations utilizing Jarzynski’s equation:

(1) $$e^{-\beta ∆G\left(x\right)}=\langle e^{-\beta W}\rangle$$

where *β* is the inverse of the thermodynamic temperature, ∆*G*(x) denotes the change in free energy along the reaction coordinate x, and W represents the non-equilibrium work associated with the reaction coordinate x during SMD simulations. A total of 10 parallel SMD simulations were performed for each system using the NAMD program to reduce the potential error in the calculation of PMF values. Furthermore, plots illustrating the variations in the potential of mean force (PMF) and force along the reaction coordinate during the dissociation process of the two inhibitors from the ATP channel were created utilizing Origin.

### 4.6. MM-GBSA (Molecular Mechanics-Generalized Born Surface Area) Calculations

The Molecular Mechanics-Generalized Born Surface Area (MM-GBSA) method strikes a balance between computational efficiency and accuracy [59,60] and is widely used to predict the binding affinity between proteins and ligands [61,62,63,64]. Additionally, MM-GBSA facilitates the analysis of energy contributions from each residue through binding free energy decomposition, identifying key interactions during the binding process [65,66]. In MM-GBSA, the calculation of the free energy for ligand–receptor binding is conducted as follows:

(2) $$∆G_{bind}=G_{complex}-\left(G_{receptor}+G_{ligand}\right)$$

(3) $$G=G_{MM}+G_{sol}-TS$$

(4) $$\Delta G_{MM}=\Delta E_{int}+\Delta E_{ele}+\Delta E_{vdw}$$

(5) $$\Delta G_{sol}=\Delta G_{GB}+\Delta G_{SA}$$

(6) $$\Delta G_{SA}=\gamma \times \Delta SASA$$

where ∆*G*_MM_, ∆*G*_sol_, and −*T*∆*S* represent changes in the gas–phase interaction energy, solvation-free energy, and configurational entropy when the protein and ligand bind. ∆*G*_MM_ includes changes in internal energy (∆*E*_int_), electrostatic energy (∆*E*_ele_), and van der Waals energy (∆*E*_vdw_). ∆*E*_int_ encompasses bond energy, angle energy, and torsional energy. ∆*G*_sol_ is composed of changes in polar solvation energy (∆*G*_GB_) and nonpolar solvation energy (∆*G*_SA_). ∆*G*_GB_ is computed by solving the GB equation [67], while ∆*G*_SA_ is estimated using the solvent-accessible surface area determined with a 1.4 Å water probe. Additionally, the surface tension constant *γ* is set to 0.0072 kcal/mol·Å^−2^, and β is set to 0 kcal/mol.

## 5. Conclusions

In this study, we conducted classic MD simulations, MM-GBSA calculations, τ-RAMD simulations, and SMD simulations to explore the binding and dissociation mechanisms between Mtb GyrB and its representative inhibitors, novobiocin and SPR719. The simulation results indicate that electrostatic interactions and van der Waals interactions are the main driving forces for the binding of inhibitors to the target. Key amino acid residues in the active pocket, such as Asn52, Asp79, Arg82, Lys108, Tyr114, and Arg141, are crucial for stabilizing the binding of inhibitors to the ATP site of GyrB. Furthermore, the binding mode suggests that the aromatic heterocyclic core of the inhibitors is essential for facilitating van der Waals interactions with hydrophobic residues within the binding pocket and forming π interactions with Arg82, Lys108, and Arg141. Additionally, the hydrogen bond interaction with Asp79 plays a critical role.

Furthermore, we investigated the dissociation behavior of GyrB inhibitors through τ-RAMD simulations and SMD simulations. The τ-RAMD results indicate that both inhibitors primarily dissociate from the ATP channel. The results from SMD simulations suggest that hydrophobic interactions with residues such as Asn53, Ile84, Pro85, Val99, and Ala113, as well as hydrogen bond interactions with residues like Glu56, Asp79, Lys108, and Arg141, play significant roles in the dissociation process. In summary, this work contributes to a deeper understanding of the binding and dissociation mechanisms between GyrB and its inhibitors, providing valuable insights for the rational design of novel GyrB inhibitors.

## Figures and Tables

**Table 1:** Binding free energies calculated by the MM-GBSA method and detailed contributions of various energy terms (kcal/mol).

Energy	3ZKB-Novobiovin	3ZKB-SPR719
Δ*E*_ele_	−48.72	−62.04
Δ*E*_vdw_	−61.13	−59.87
Δ*E*_MM_ ^a^	−109.85	−121.91
Δ*G*_SA_	−8.28	−6.53
Δ*G*_GB_	72.26	68.51
Δ*G*_sol_ ^b^	63.98	61.98
Δ*G*_polar_ ^c^	23.54	6.47
Δ*G*_nonpolar_ ^d^	−69.41	−66.4
Δ*H*_bind_	−52.5	−59.95
−*T*Δ*S*	28.16	33.14
Δ*G*_bind_	−24.34	−26.81
Ki (nM)	13	9
Δ*G*_exp_ ^e^	−10.82	−11.04

^a^ $\Delta E_{MM}=\Delta E_{ele}+\Delta E_{vdw}.$ ^b^ $\Delta G_{sol}=\Delta G_{GB}+\Delta G_{SA}.$ ^c^ $\Delta G_{polar}=\Delta E_{ele}+\Delta G_{GB}.$ ^d^ $\Delta G_{nonpolar}=\Delta E_{vdw}+\Delta G_{SA}.$ ^e^ Calculated by the experimental Ki according to the equation Δ*G*_exp_ = *RT* × ln (Kd) ≈ *RT* × ln (Ki), where T = 300 K.

**Table 2:** Hydrogen bond occupancy between GyrB and novobiocin, SPR719.

Complex	Donor	Acceptor	Distance (Å)	Angel (°)	Occupancy (%)
3ZKB_novobiocin	ligand@O11-H30	ASP_79@OD1	2.5876	168.1364	99.91
	ligand@O5-H11	GLY_107@O1479	2.6696	165.8257	99.22
	ligand@N1-H17	PHE_109@O1521	2.8626	161.4976	63.76
	HIE_89@NE2-HE2	ligand@O5	2.8912	154.5943	25.39
	ASN_52@ND2-HD2	ligand@O9	2.8714	160.222	13.12
	GLY_107@NH	ligand@O6	2.8935	143.3523	11.15
3ZKB_SPR719	ligand@N6-H20	ASP_79@OD1	2.782	158.5813	76.02
	ligand@N3-H12	ASP_79@OD1	2.7976	159.6323	74.85
	ARG_141@NH1-HH12	ligand@N4	2.8998	148.7218	35.25
	ARG_141@NH2-HH22	ligand@O2	2.8837	162.1836	27.17
	ligand@N6-H20	ASP_79@OD2	2.7771	158.7187	20.58
	ligand@N3-H12	ASP_79@OD2	2.7982	159.7471	20.52

## Data Availability

Data are contained within the article and supplementary materials.

## Supplementary Materials

The following supporting information can be downloaded at: https://www.mdpi.com/article/10.3390/ijms25073764/s1.
