# Supplementary material for: Revealing the Interaction Mechanism between Mycobacterium tuberculosis GyrB and Novobiocin, SPR719 through Binding Thermodynamics and Dissociation Kinetics Analysis
Source: Int J Mol Sci. 2024 Mar 28;25(7):3764. doi: 10.3390/ijms25073764 (PMC11011931; doi:10.3390/ijms25073764)
Supplement: Supplementary file 1 [file ijms-25-03764-s001.zip › ijms-2906918-supplementary.pdf]

Supplementary Material

Revealing the Interaction Mechanism between Mycobacterium tuberculosis GyrB and Novobiocin, SPR719 through binding Thermodynamics and Dissociation Kinetics analysis

Xiaofei Qiu[1,#], Qianqian Zhang[2,#], Zhaoguo Li[1], Juan Zhang[1], Huanxiang Liu[2]*

[1]School of Pharmacy, Lanzhou University, Lanzhou 730000, China

[2]Faculty of Applied Science, Macao Polytechnic University, Macao, SAR, China

# These authors contributed equally.

* Corresponding author

Tel.: +853-8599-6874

E-mail address: hxliu@mpu.edu.mo

Table S1. Cluster analysis of conformations in the two systems.

Systems	Populations（%）		
	Cluster1(%)	Cluster2(%)	Cluster3(%)
GyrB-novobiocin	83.6	10.9	5.5
GyrB-SPR719	90.0	9.1	0.9

Table S2. Statistical analysis of the number of dissociation pathways predicted by τ-RAMD for the two compounds.

Ligand	magnitude of random force (kcal/mol)	path1	path2
Novobiocin	25	97	3
SPR719	25	98	2

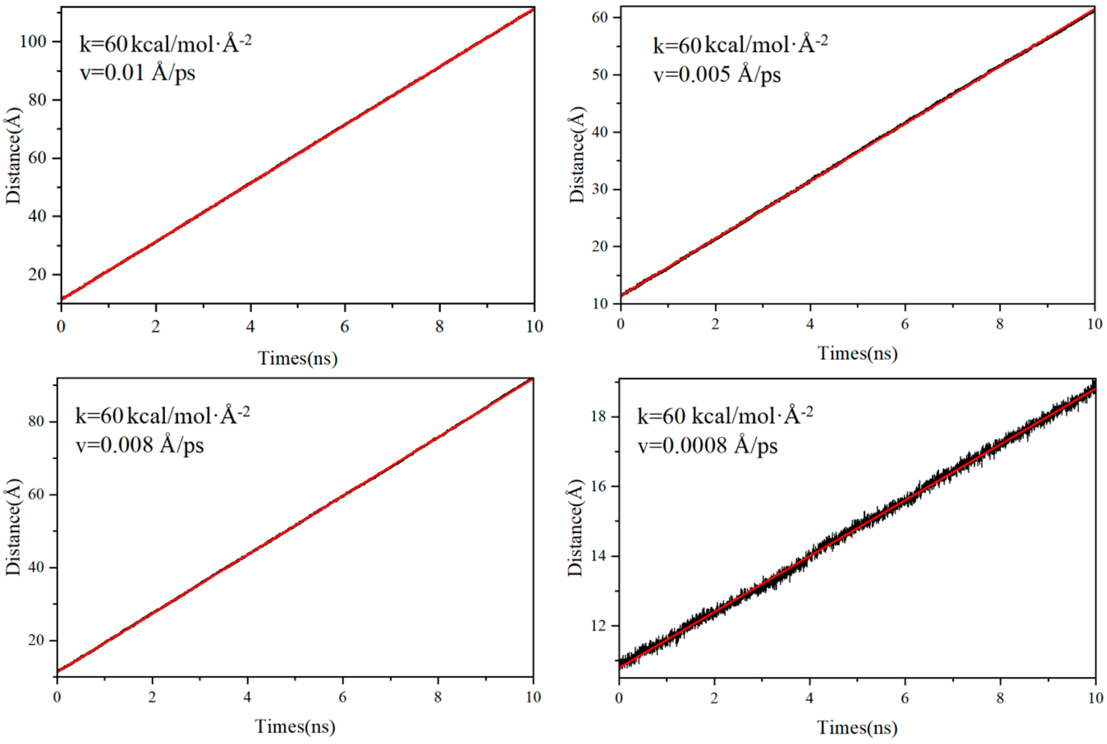

Figure S1. SMD parameter calibration. This figure shows the changes in reaction coordinates over time for different values of v (0.01, 0.005, 0.008, and 0.0008 Å/ps), while keeping the elastic constant k = 60 kcal/mol•Å^{-2} constant.

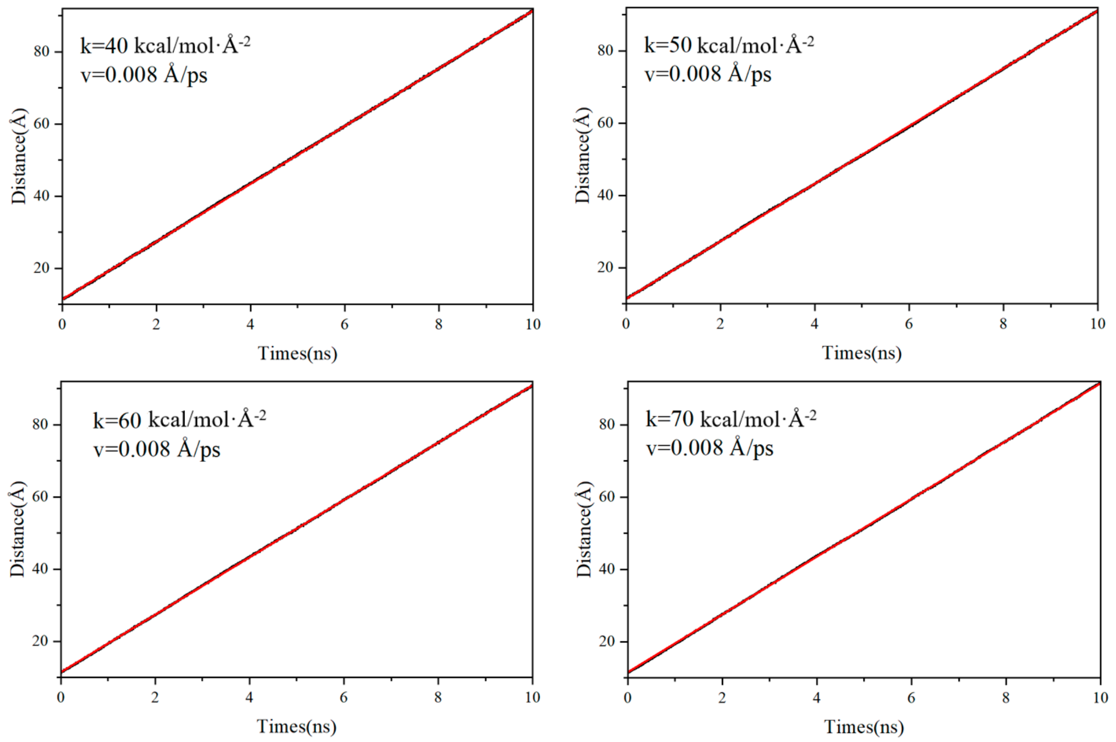

Figure S2. SMD parameter calibration. This figure shows the changes in reaction coordinates over time for different values of k (40, 50, 60, 70 kcal/mol•Å^{-2}), while keeping the stretching velocities v = 0.008 Å/ps constant.

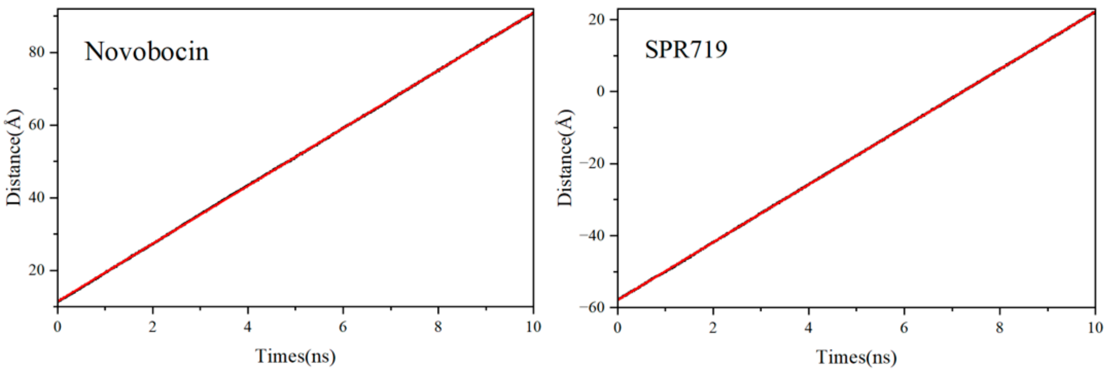

Figure S3. Distance between ligands and the center of the binding pocket over simulation time for the two systems with an elastic constant of 60 kcal/mol•Å^{-2} and a stretching velocity of v = 0.008 Å/ps.
